# *EGFR*突变状态对Ⅲ期非鳞非小细胞肺癌患者放化疗近期疗效和长期生存的预测价值

**DOI:** 10.3779/j.issn.1009-3419.2011.09.03

**Published:** 2011-09-20

**Authors:** 付海 李, 桦 白, 夏南 李, 梅娜 吴, 荣 余, 安辉 石, 丽 尹, 洁 王, 广迎 朱

**Affiliations:** 1 100142 北京，北京大学临床肿瘤学院，北京肿瘤医院暨北京市肿瘤防治研究所，恶性肿瘤发病机制及转化研究教育部重点实验室，放射治疗科 Department of Radiation Oncology, Key Laboratory of Carcinogenesis and Translational Research (Ministry of Education), Peking University School of Oncology, Beijing Cancer Hospital & Institute, Beijing 100142, China; 2 100142 北京，北京大学临床肿瘤学院，北京肿瘤医院暨北京市肿瘤防治研究所，恶性肿瘤发病机制及转化研究教育部重点实验室，胸部肿瘤内科 Department of Thoracic Medical Oncology, Key Laboratory of Carcinogenesis and Translational Research (Ministry of Education), Peking University School of Oncology, Beijing Cancer Hospital & Institute, Beijing 100142, China

**Keywords:** 肺肿瘤, 放化疗, EGFR, 突变, Lung neoplasms, Chemoradiotherapy, EGFR, Mutation

## Abstract

**背景与目的:**

表皮生长因子受体（epidermal growth factor receptor, EGFR）突变状态与Ⅲ期非小细胞肺癌放化疗近期疗效和生存的关系是目前临床研究热点。本研究旨在探讨*EGFR*突变状态与Ⅲ期非鳞非小细胞肺癌放化疗疗效的关系。

**方法:**

本研究共计入组187例Ⅲ期非鳞非小细胞肺癌患者，其中87例能够评估放化疗近期疗效和2年生存率，128例患者适合评估一线化疗疗效。采用变性高效液相色谱法检测*EGFR*基因突变状态。

**结果:**

*EGFR*突变阳性患者对联合放化疗的客观缓解率为84.6%（33/39），明显高于*EGFR*突变阴性患者56.3%（27/48）（*P*=0.004）。2年生存率*EGFR*突变阳性患者为53.8%（21/39），*EGFR*突变阴性患者为50%（24/48），两组在长期生存方面无统计学差异（*P*=0.871）。

**结论:**

在Ⅲ期非鳞非小细胞肺癌中，*EGFR*突变预示更高的放化疗近期疗效，与生存期无关。

## Abstract (en)

**Background and objective:**

The presence of epidermal growth factor receptor (EGFR) mutations is predictive of a better response to EGFR tyrosine kinase inhibitors (EGFR-TKIs) and initial chemotherapy in advanced non-squamous non-small cell lung cancer (NSCLC). The aim of this study is to analyze the association between the *EGFR* mutations and the outcome of combined chemoradiotherapy in stage Ⅲ non-squamous NSCLC.

**Methods:**

Patients with stage Ⅲ non-squamous NSCLC whose *EGFR* mutation status had been identified were retrospectively analyzed. The response of 87 patients to combined chemoradiotherapy, the two-year survival rate, and the response of 128 patients to initial chemotherapy were evaluated.

**Results:**

The response rate to combined chemoradiotherapy was 84.6% (33/39) in *EGFR* mutation-positive patients, significantly higher than the 56.3% (27/48) response rate in *EGFR* mutation-negative patients (*P*=0.004). Two-year survival rates were 53.8% and 50% in *EGFR* mutation-positive and mutation-negative patients, respectively. There was no significant difference in the overall survival for both patient groups. The overall response rate to initial chemotherapy was 34.5% (19/55) in *EGFR* mutation-positive patients, compared with the 21.9% (16/73) response rate in *EGFR* mutation-negative patients.

**Conclusion:**

*EGFR* mutation-positive status can predict better response to combined chemoradiotherapy, but is not associated with overall survival in patients with stage Ⅲ non-squamous NSCLC.

在世界范围肺癌是首位的癌症致死原因^[1]^，在中国肺癌的发病率和死亡率也均居首位^[2]^。肺癌包括非小细胞肺癌（non-small cell lung cancer, NSCLC）和小细胞肺癌两大类，其中85%为NSCLC^[3-5]^。传统上NSCLC包括鳞癌和非鳞癌（non-squamous carcinoma, NSC），NSC又包括腺癌、大细胞癌及其它类型^[6]^，约占肺癌总数的50%-60%^[5]^。

联合放化疗是当前不可切除的Ⅲ期NSCLC的主要治疗手段，与序贯放化疗相比，同步放化疗的有效率更高、生存期更长^[7]^。研究^[8-10]^显示表皮生长因子受体酪氨酸激酶抑制剂（epidermal growth factor receptor-tyrosine kinase inhibitors, EGFR-TKIs）对伴有*EGFR*激活突变（外显子19缺失和外显子21突变）的晚期NSC患者有效率更高，*EGFR*突变的晚期肺腺癌患者对一线化疗的有效率更高^[8]^。

对于Ⅲ期NSC患者*EGFR*突变是否与放化疗近期疗效和长期生存相关，目前尚不清楚。本研究旨在探讨*EGFR*突变状态与联合放化疗近期疗效和长期生存的关系。

## 1 材料与方法

### 1.1 患者资料

对187例*EGFR*突变状态明确的Ⅲ期NSC患者进行回顾性分析。其中87例患者可评估放化疗疗效和2年生存率，患者均接受了含铂方案化疗及同步或序贯胸部放疗，同时满足以下入选标准：病理学证实的NSC，有可测量的病灶，一般状态评分0分-1分，≤75岁，无不可控制的糖尿病或其它严重的疾病或并发症。128例患者适合评估一线化疗疗效。

### 1.2 *EGFR*突变检测

报道^[11]^显示循环血液中存在肿瘤细胞来源的游离DNA。本研究检测标本为外周血游离DNA或肿瘤组织，*EGFR*突变检测采用变性高效液相色谱分析法^[12]^。

### 1.3 评估方法

疗效评价采用实体瘤疗效评价标准^[13]^。总生存期定义为自病理确诊日期至死亡或末次随访。*EGFR*突变定义为外显子19缺失突变和/或外显子21置换突变，不含这两种突变者定义为*EGFR*野生型。

### 1.4 统计学分析

采用SPSS软件进行统计分析，率的比较采用卡方检验。采用*Logistic*回归多因素分析评价放化疗疗效和基线特征之间的关系。采用*Kaplan-Meier*法绘制生存曲线并接受*Log-rank*检验。*P* < 0.05为差异具有统计学意义。

## 2 结果

### 2.1 患者资料

截至2011年1月，共有187例Ⅲ期非鳞非小细胞肺癌患者*EGFR*突变状态明确，*EGFR*总突变率为40.6%（76/187），女性（46.2%, 42/91）高于男性（35.4%, 34/96），不吸烟者（45%, 49/109）高于吸烟者（34.6%, 27/78），体重下降 < 5%者（43.7%, 73/167）高于体重下降≥5%者（15%, 3/20）。其中87例患者能够评估放化疗疗效和2年生存率，*EGFR*突变患者39例，*EGFR*野生型患者48例（表 1）。128例患者初始治疗采用含铂方案化疗，其中*EGFR*突变患者55例，*EGFR*野生型患者73例。

**1:** 87例放化疗患者一般资料 Characteristics in patients received chemoradiotherapy

Characteristic	Mutation-positive *EGFR*	Mutation-negative *EGFR*
No. of patients	39	48
Age (yr)		
≥65	9 (23.1%)	11 (22.9%)
< 65	30 (76.9%)	37 (77.1%)
Gender		
Female	17 (43.6%)	17 (35.4%)
Male	22 (56.4%)	31 (64.6%)
Smoking		
Never	23 (59.0%)	26 (54.2%)
Ever	16 (41.0%)	22 (45.8%)
Performance status		
0	5 (12.8%)	10 (20.8%)
1	34 (87.2%)	38 (79.2%)
Weight loss		
≥5%	1 (2.6%)	7 (14.6%)
< 5%	38 (97.4%)	41 (85.4%)
Clinical stage		
Ⅲa	7 (17.9%)	7 (14.6%)
Ⅲb	32 (82.1%)	41 (85.4%)
Chemoradiation		
Concurrent	26 (66.7%)	25 (52.1%)
Sequential	13 (33.3%)	23 (47.9%)
Radiation dose		
60 Gy-70 Gy	31 (79.5%)	39 (81.2%)
40 Gy-59 Gy	8 (20.5%)	9 (18.8%)
EGFR-TKIs		
Yes	16 (41.0%)	13 (27.1%)
No	23 (59.0%)	35 (72.9%)
EGFR: epidermal growth factor receptor; EGFR-TKIs: EGFR tyrosine kinase inhibitors.		

**2:** 放化疗近期疗效 Response to chemoradiotherapy

Response	Mutated *EGFR*	Wild-type *EGFR*
No. of patients	39	48
CR	3 (7.7%)	1 (2.1%)
PR	30 (76.9%)	26 (54.2%)
ORR	33 (84.6%)	27 (56.3%)
SD	1 (2.6%)	14 (29.2%)
PD	5 (12.8%)	7 (14.6%)
CR: complete response; PR: partial response; ORR: overall response rate; SD: stable disease; PD: progressive disease.		

### 2.2 疗效

*EGFR*突变患者对联合放化疗的客观缓解率为84.6%（33/39），明显高于*EGFR*野生型患者（56.3%, 27/48）（*P*=0.004）。包括*EGFR*突变状态、吸烟史、性别、年龄、一般状况评分、体重下降、临床分期、放化疗模式、放疗剂量为协变量的多因素分析显示，*EGFR*突变（*P*=0.004）、吸烟（*P*=0.024）、性别（*P*=0.039）为独立的放化疗疗效预测因素。

### 2.3 长期生存

末次随访于2011年1月进行，中位随访时间为25.1（2.3-60）个月，统计学分析亦同时进行。中位生存时间*EGFR*突变阳性患者为25（4.6-60）个月，*EGFR*突变阴性患者为24.5（2.3-58.4）个月；1年、2年生存率*EGFR*突变阳性患者为84.6%、53.8%，*EGFR*突变阴性患者为85.4%、50%，两组相比无统计学差异（*P*=0.871）（图 1）。

**1:**
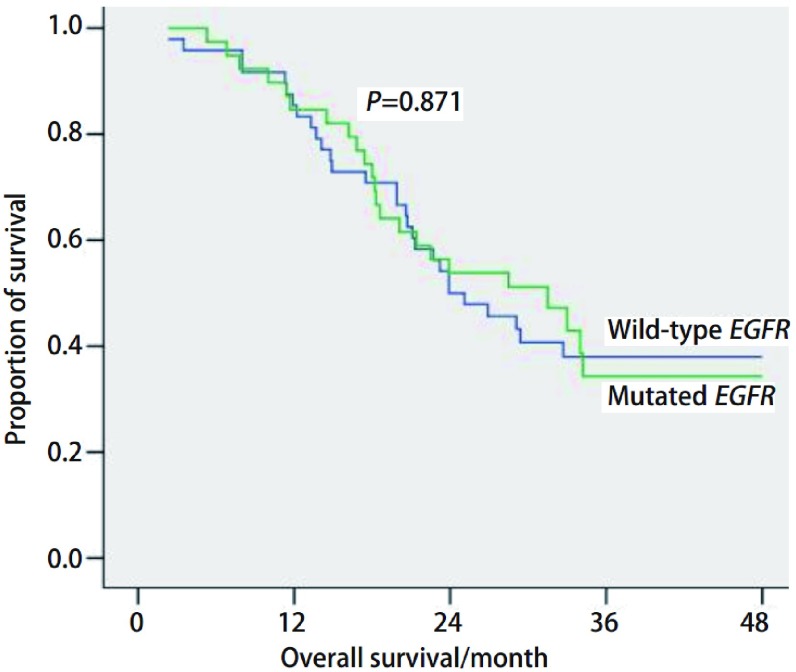
生存曲线（突变型患者39例，野生型患者48例） Overall survival (Mutated *EGFR*: *n*=39, Wild-type *EGFR*: *n*=48)

### 2.4 放化疗后EGFR-TKIs治疗

共有29例放化疗后疾病进展（progressive disease, PD）的患者接受过EGER-TKIs治疗，其中*EGFR*突变型患者16例，近期疗效为部分缓解（partial response, PR）1例，稳定（stable disease, SD）8例，PD 7例。野生型患者13例，SD 6例，PD 7例。接受EGFR-TKIs治疗的患者与未接受EGFR-TKIs治疗的患者相比，长期生存无统计学差异（*P*=0.271）。

## 3 讨论

本研究显示在Ⅲ期NSC患者中*EGFR*突变意味着更高的放化疗近期疗效，但与长期生存无关。

*EGFR*突变预示着更高的放化疗近期疗效，本研究发现*EGFR*突变型患者对联合放化疗和初治含铂方案化疗的有效率均高于*EGFR*野生型患者。关于*EGFR*突变状态与Ⅲ期NSC联合放化疗近期疗效的关系尚未见报道。*EGFR*野生型患者对联合放化疗的有效率与几项有关局部晚期NSCLC的研究报道^[14-16]^类似。IPASS研究^[8]^显示晚期肺腺癌患者对一线PC方案化疗的有效率在突变组高于未突变组（47.3% *vs* 23.5%）。本研究发现两组间虽然近期疗效存在明显差别，但长期生存不存在明显差异。对于*EGFR*突变的以腺癌为主的晚期NSCLC，多项关于一线EGFR-TKI与化疗对比的随机研究^[8, 9, 17]^也有类似发现：近期有效率差别明显而长期生存相似。我们推测*EGFR*激活突变使肿瘤细胞增殖速度更快，一方面其对治疗（包括EGFR-TKIs、化疗、放疗）的反应更敏感，表现为更高的近期疗效；另一方面*EGFR*激活突变使肿瘤细胞更具侵袭性，更容易发生转移，一旦进展可能发展更为迅速，从而抵消了近期疗效的优势。

*EGFR*突变型和野生型患者放化疗后疾病进展后使用EGFR-TKIs的有效率分别为1/16、0/13。生存分析显示放化疗后出现疾病进展的患者使用EGFR-TKIs并未带来生存受益（*P*=0.271）。SWOG S0023^[18]^是一项针对Ⅲ期NSCLC患者经EP方案同步放化疗+多西他赛巩固治疗后进行吉非替尼或安慰剂维持治疗的Ⅲ期临床研究，此研究没有对入选患者的*EGFR*突变状态进行筛查，结果显示吉非替尼组和安慰剂组的中位生存期分别为23个月（*n*=118）和35个月（*n*=125）（*P*=0.013），吉非替尼维持治疗组生存期更短。我们推测联合放化疗可能会改变EGFR信号传导通路，进而影响后续EGFR-TKIs的疗效，此推论尚需进一步研究论证。

本研究显示在Ⅲ期非鳞非小细胞肺癌中，*EGFR*突变预示着更高的放化疗近期疗效，与长期生存无关。

## References

[1] Parkin DM, Bray F, Ferlay J (2005). Global cancer statistics, 2002. CA Cancer J Clin.

[b2] 2Zhao P, Chen WQ, ed. Chinese Cancer Registry Annual Report, 2009. 1st ed. Beijing: Military Medical Sciences Press, 2010. 626-628.赵平, 陈万青主编. 2009中国肿瘤登记年报. 第一版. 北京: 军事医学科学出版社, 2010. 626-628.

[3] Yang P, Allen MS, Aubry MC (2005). Clinical features of 5, 628 primary lung cancer patients: experience at Mayo Clinic from 1997 to 2003. Chest.

[4] Govindan R, Page N, Morgensztern D (2006). Changing epidemiology of small-cell lung cancer in the United States over the last 30 years: analysis of the surveillance, epidemiologic, and end results database. J Clin Oncol.

[5] Youlden DR, Cramb SM, Baade PD (2008). The International Epidemiology of Lung Cancer: Geographical distribution and secular trends. J Thorac Oncol.

[6] Beasley MB, Brambilla E, Travis WD (2005). The 2004 world health organization classification of lung tumors. Semin Roentgenol.

[7] Anne A, Cecile LP, Estelle R (2010). *Meta*-analysis of concomitant versus sequential radiochemotherapy in locally advanced non-small-cell lung cancer. J Clin Oncol.

[8] Mok TS, Wu YL, Thongprasert S (2009). Gefitinib or carboplatin-paclitaxel in pulmonary adenocarcinoma. N Engl J Med.

[9] Maemondo M, Inoue A, Kobayashi K (2010). Gefitinib or chemotherapy for non-small-cell lung cancer with mutated *EGFR*. N Engl J Med.

[10] Douillard JY, Shepherd FA, Hirsh V (2010). Molecular predictors of outcome with gefitinib and docetaxel in previously treated non-small-cell lung cancer: data from the randomized phase Ⅲ INTEREST trial. J Clin Oncol.

[11] Maheswaran S, Sequist LV, Nagrath S (2008). Detection of mutations in *EGFR* in circulating lung-cancer cells. N Engl J Med.

[12] Hua B, Li M, Shuhang W (2009). Epidermal growth factor receptor mutations in plasma DNA samples predict tumor response in Chinese patients with stages ⅢB to Ⅳ non-small-cell lung cancer. J Clin Oncol.

[13] Therasse P, Arbuck SG, Eisenhauer EA (2000). New guidelines to evaluate the response to treatment in solid tumors. J Natl Cancer Inst.

[14] Vokes EE, Herndon JE 2nd, Kelley MJ (2007). Induction chemotherapy followed by chemoradiotherapy compared with chemoradiotherapy alone for regionally advanced unresectable stage Ⅲ non-small-cell lung cancer: cancer and leukemia group B. J Clin Oncol.

[15] Divers SG, Spencer SA, Carey D (2005). Phase Ⅰ/Ⅱa study of cisplatin and gemcitabine as induction chemotherapy followed by concurrent chemoradiotherapy with gemcitabine and paclitaxel for locally advanced non-small-cell lung cancer. J Clin Oncol.

[16] Vokes EE, Herndon JE 2nd, Crawford J (2002). Randomized phase Ⅱ study of cisplatin with gemcitabine or paclitaxel or vinorelbine as induction chemotherapy followed by concomitant chemoradiotherapy for stage ⅢB non-small-cell lung cancer: cancer and leukemia group B study 9431. J Clin Oncol.

[17] Mitsudomi T, Morita S, Yatabe Y (2010). Gefitinib versus cisplatin plus docetaxel in patients with non-small-cell lung cancer harbouring mutations of the epidermal growth factor receptor (WJTOG3405): an open label, randomised phase 3 trial. Lancet Oncol.

[18] Kelly K, Chansky K, Gaspar LE (2008). Phase Ⅲ trial of maintenance gefitinib or placebo after concurrent chemoradiotherapy and docetaxel consolidation in inoperable stage Ⅲ non-small-cell lung cancer: SWOG S0023. J Clin Oncol.

